# Beyond the Transcripts: What Controls Protein Variation?

**DOI:** 10.1371/journal.pbio.1001146

**Published:** 2011-09-06

**Authors:** Laura Straub

**Affiliations:** Freelance Science Writer, Arlington, Virginia, United States of America

**Figure:**
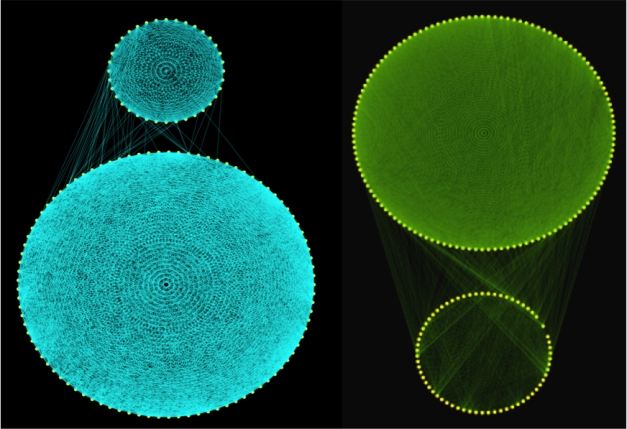
Proteins involved in two biological processes are tightly co-regulated, as are their corresponding transcripts. Remarkably, the proteins do not seem to care about what the transcripts are doing and form networks according to their own rules.

It was long thought that if you knew the contents of the genome, and which genes made which proteins, you would know the basic instructions for building an organism and all the processes it needs to stay alive. Organisms convert extensive genomic instructions into a specific protein repertoire via transcription and translation. It's clear that the act of transcription is tightly controlled by the cell, and scientists have a relatively good grasp of the mechanisms that regulate gene expression. As a result, we tend to have a rather “transcript-centric” view of biology, assuming that more transcript automatically translates into more proteins. This is the tacit premise underlying countless gene-transcription studies in which levels of thousands of transcripts are measured under a range of conditions and the results happily extrapolated to their protein products. Notionally, however, this is not necessarily the case, because downstream controls on translation, protein folding, and protein degradation have the potential to trump the impact of earlier transcriptional activity.

While previous studies in yeast have demonstrated instances where protein levels do correspond with transcript levels, they tended to focus on the relationship between transcript and protein levels for different genes within a single individual, demonstrating that low-abundance proteins tend to have low-abundance transcripts and the contrary for high-abundance proteins. However, these findings do not necessarily imply that there will be a good correlation between transcript and protein levels for the *same* genes across *different* individuals, an issue that is critical in using transcript levels for biomarker discovery. In a new study published in *PLoS Biology*, Antonio Bedalov and colleagues investigated the latter problem using genetically distinct yeast strains. They measured the extent to which variation seen in protein levels across these distinct strains could be explained by corresponding levels of variation in the underlying transcripts and found overwhelming evidence to the contrary. Instead, their results point to regulatory mechanisms unrelated to transcriptional control as the ultimate determiners of proteome variation in a genetically diverse population.

To explore the relationship between transcripts and corresponding protein levels, the researchers used highly sensitive methods to simultaneously measure levels of proteins and their transcripts in 95 genetically distinct yeast strains. Using an enhanced version of an algorithm they had previously developed to measure protein levels, they reanalyzed an existing mass spectrometry–based dataset and obtained more accurate and detailed results for 354 high-abundance proteins—twice as many as they had previously measured. Of note, the corresponding transcripts for these 354 proteins were among those with the most variable level between individuals, an important feature since this highly variant group of transcripts is most likely to influence protein level through their abundance.

The researchers used statistical techniques to identify proteins that might be co-regulated, that is, controlled by the same genetic switches, and found two groups of highly co-regulated proteins: one associated with making amino acids and one associated with the ribosomes.

They then measured the extent to which underlying transcript levels correlated with protein concentrations among these two identified co-regulated networks. They found unexpectedly low correlation, with proteins and transcripts well-correlated for only 27% of the genes, suggesting that other, post-transcriptional, mechanisms must account for much of the variation seen.

To explore the underlying genetic forces that might account for the differences, the researchers mapped out correlations between locations in the DNA sequence that differed between the yeast strains and the levels of proteins and/or their transcripts. This should flag up between-strain genetic differences that are responsible for the observed regulatory differences, whether they operate at the level of the transcript or the protein. They found that the vast majority of influential genetic variants acted to determine *either* transcript levels *or* protein levels; rarely both. Post-transcriptional genetically determined variation was apparently an independent dominant force in shaping each strain's proteome.

Taken together, these results indicate that variation in underlying transcript levels cannot account for the majority of variation observed in the corresponding protein levels between yeast strains. Rather, the signals controlling whether a gene is ultimately translated into a working protein operate through mechanisms that come into play *after* the genes are transcribed. Future studies, aided by advances in high-throughput protein analysis, will help identify which transcription-independent mechanisms are responsible for protein level variations in yeast, and whether such factors operate in other organisms as well. And since proteins, in the end, not genes, more directly account for the way organisms look, function, and behave, such questions will eventually help us understand the forces underlying the diversity of life from yeast to humans.

**Foss EJ, Radulovic D, Shaffer SA, Goodlett DR, Kruglyak L, et al. (2011) Genetic variation shapes protein networks mainly through non-transcriptional mechanisms. doi:10.1371/journal.pbio.1001144**

